# Hereditary angioedema: New therapeutic options for a potentially deadly disorder

**DOI:** 10.1186/1471-2326-10-3

**Published:** 2010-05-14

**Authors:** Frank J Eidelman

**Affiliations:** 1 Department of Allergy and Immunology, Medical Informatics, Cleveland Clinic Florida, 2950 Cleveland Clinic Blvd, Weston, FL 33331, USA

## Abstract

Although the biochemistry of hereditary angioedema (HAE) is fairly well understood today, the lag in diagnosis of a decade or more suggests that clinicians have low awareness of this disease. This lag in diagnosis and hence treatment certainly stems from the rarity and complexity of the presentation which can be easily mistaken for allergic and non-allergic reactions alike. The symptoms of the disease include acute swelling of any or multiple parts of the body. The attacks may be frequent or rare, and they may vary substantially in severity, causing stomach discomfort or periorbital swelling in mild cases and hypovolemic shock due to abdominal fluid shift or asphyxiation in the most severe cases. Given that these patients are at significant risk for poor quality of life and death, greater awareness of this disease is needed to ensure that newly available, effective medications are used in these patients. These new medications represent significant advances in HAE therapy because they are targeted at the plasma cascades implicated in the pathophysiology of this disease. The clinical presentation of HAE, overlapping symptoms with other angioedemas, and available therapies are reviewed.

## Introduction

In 1888, Sir William Osler provided a medical description of angioedema (AE) that distinguished an inherited form of the disease[1]. His description was the first to provide full clinical details. Seventy-five years later, Donaldson and Evans described patients with clinical features similar to those first described by Osler. The authors demonstrated a deficiency of C1 esterase inhibitor in the blood of these patients, although the extent of the deficiency was not able to be determined. Today, the biochemistry of the disease is better understood, but clinicians have low awareness of hereditary AE (HAE) and other types of non-allergic AE. Therefore, HAE is frequently undiagnosed. Approximately 50% of patients will be symptomatic by age 10[2]. However, accurate diagnosis may be delayed by decades[2]. For many, the disease results in multiple emergency department visits per year,[3] and for those patients with abdominal symptoms, one third may be subjected to inappropriate medical intervention due to misdiagnosis[4]. The fear of death from asphyxiation is an unfortunate but common part of life for many patients with HAE because of the risk of laryngeal swelling.

New medications are available for prophylaxis and acute treatment of patients with HAE. These new medications offer targeted actions that reduce the potential for long-term adverse effects, such as those experienced with long-term androgen exposure, and they have quick onset action, making them the best choice for acute treatment. Increased awareness of HAE symptoms, of available diagnostics, and of available treatments among clinicians will help reduce time to diagnosis and improve disease management. The following discussion will help clinicians identify HAE and choose among available therapies.

## Mechanism of disease

HAE affects 1:10,000 to 1:50,000 people and is caused by mutations in the C1 inhibitor gene[5]. The C1 inhibitor gene is located on chromosome 11, and multiple mutations resulting in C1 inhibitor deficiency have been identified. Since C1 inhibitor has a broad inhibitory role, C1 inhibitor deficiency affects the regulation of multiple plasma cascade pathways, namely the contact, fibrinolytic, and complement pathways. C1 inhibitor binds irreversibly in these pathways. Therefore, new C1 inhibitor molecules are needed to maintain homeostasis once the substrate is bound. However, people with HAE have C1 inhibitor levels <50% of normal, and they cannot produce C1 inhibitor fast enough to offset consumption in activated pathways[6]. Massive bradykinin release via the contact pathway is thought to be the primary cause of symptoms in both HAE and acquired AE[7,8]. Therefore, the contact pathway has been a target of therapeutic investigation because C1 inhibitor blocks the activity of factor XII and kallikrein in this pathway[6]. Since plasma kallikrein releases bradykinin from high-molecular-weight kininogen, its inhibition has also been a target of investigation[6].

Approximately 85% of HAE cases are type 1, a deficiency in the amount of C1 inhibitor produced. Nearly all other cases are type 2 HAE, which is characterized by the expression of dysfunctional C1 inhibitor. Inherited C1 inhibitor deficiency with normal C1 inhibitor activity (formerly HAE type 3) is a very rare disorder seen primarily in women and may be caused by increased activity of factor XII within the contact pathway, leading to increased bradykinin and angioedema[2,9]. Although HAE is primarily an autosomal dominant inherited disease, it appears de novo in approximately 25% of cases due to spontaneous mutations.

## Presentation and differential diagnosis

Although no clear bias for sex or race has been observed for HAE, it appears to affect women more severely due to fluctuating estrogen levels[10]. Evidence for estrogen sensitivity comes from reports of worsening symptoms while using estrogen-containing oral contraceptives, but not progesterone-only oral contraceptives[2,11].

Trauma and stress are the most commonly reported triggers for HAE attacks[12]. In many cases however, there is no obvious trigger. Swelling episodes due to HAE typically worsen over one to two days and then resolve within an additional two days. More severe attacks may involve 5 days of unremitting symptoms[13]. Any individual part of the body or multiple sites may be affected[8]. Patients may experience prodromal symptoms that include tingling or burning in the area of an imminent attack[9]. Erythema marginatum, a serpiginous, non-pruritic rash may appear on the trunk and appendages as part of the prodrome in one-third of patients[9]. Inflammation and allergy are not implicated in HAE, and uritcaria is noteably absent during attacks[14].

More than 90% of patients will experience abdominal HAE attacks[12]. In most patients, the abdomen will be protuberant and tender[10]. Bowel sounds will vary between inactive and hyperactive, and guarding and rebound tenderness may be present[10]. The pain associated with abdominal angioedema may have a severe acute onset or recur as chronic abdominal pain of moderate severity[6]. Painful abdominal cramps are rated as severe to excruciating in 87% of patients[15]. In addition, 78% of patients report vomiting, and 65% report diarrhea with abdominal pain[15]. Constipation and intestinal obstruction may also occur[6,12].

Abdominal pain may occur for many years in the absence of cutaneous symptoms, but this is a rare occurrence[16]. HAE abdominal symptoms can be mistaken for other causes of abdominal pain, such as acute appendicitis[5]. Attacks are frequently accompanied by prodromal symptoms, including nonspecific complaints of irritability, aggressiveness, fatigue, or hunger[15]. Substantial fluid accumulation in the intestinal wall and lumen and in the peritoneal cavity will cause intestinal wall swelling and ascites. In rare cases, hypovolemic shock may occur due to the volume of fluid migration[16]. Patients with abdominal HAE symptoms may require narcotic analgesics for pain relief, and those at risk for hypovolemic shock will require aggressive rehydration.

Facial attacks will affect approximately half of all patients with HAE[6]. Facial attacks may involve severe swelling, and they carry significant risk of asphyxiation from extension to the larynx[17]. In the event of laryngeal involvement, intubation or tracheotomy may be required to prevent asphyxiation[12].

More than 95% of patients with HAE will experience extremity attacks[12]. Urogenital attacks are common as well[8]. Both types of attacks can be triggered by normal activity and may cause enough discomfort to require treatment.

In the absence of treatment, a patient with HAE may experience weekly attacks[9]. Others may have symptom-free periods of a year or more[18]. Although causes include physical trauma, surgical/medical/dental procedures, repetitive daily activities, prolonged standing, infection, emotional stress, and certain medications, often the cause for an attack cannot be identified[2,8,10,12].

**Figure 1:**
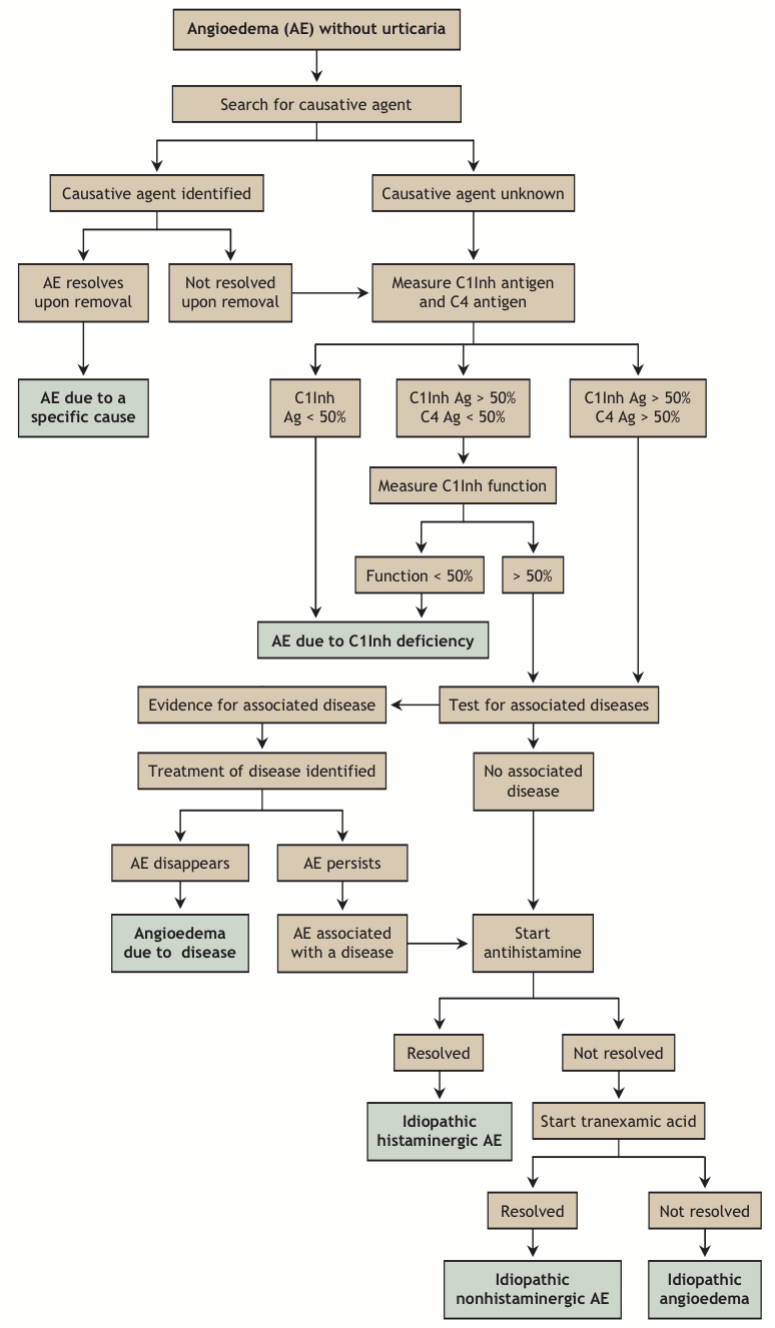
**HAE differential diagnostic algorithm**. C4 complement protein levels can be used as a screening test for HAE. Low C1 inhibitor levels support an HAE diagnosis in the absence of a family history since 25% of HAE cases are the result of spontaneous mutations in individuals. Reproduced with permission from Zingale et al.[35]

During an attack, the clinical symptoms of HAE will include non-pitted, non-pruritic subcutaneous or submucosal edema, with a possible non-pruritic serpentine erythematous rash, and may include multiple sites of swelling[9]. Patients may also experience burning and pain at the affected site. The extent of C1 inhibitor deficiency is not related to symptom severity or to the bodily location of an attack[19]. A diagnostic algorithm is provided to help rule out causative agents and diseases and to guide laboratory study interpretation (Figure 1).

A thorough patient history and laboratory evaluation will aid in the differential diagnosis. In 75% of cases, a thorough patient history will reveal recurrent swelling episodes without urticaria, a family history of these episodes, symptom emergence around age 10, and more severe symptoms starting at puberty[9,10]. Laboratory markers that will help clinicians distinguish among AEs appear in Table 1. C4 complement levels are typically low in HAE, whereas C1q and C3 are typically normal[9]. Normal C4 levels during periods of wellness does not preclude the HAE diagnosis, and repeat values should be measured during an acute attack when they are depressed. Testing for C1 inhibitor antigenic and functional levels provides for a definitive diagnosis and helps distinguish between HAE types 1 and 2[9].

**Table 1:** C1 inhibitor levels and complement protein concentrations in angioedemas [6].

Angioedema Type	C1 inhibitor	Functional C1 inhibitor	C4	C3	C1q
**HAE type 1**	<30%	<30%	Low	Normal	Normal

**HAE type 2**	Normal/High	<30%	Low	Normal	Normal

**Inherited angioedema with normal C1 inhibitor**	Normal	Normal	Normal	Normal	Normal

**Acquired**	Low	Low	<30%	Normal/Low	Normal/Low

**Angiotensin converting enzyme inhibitor induced**	Normal	Normal	Normal	Normal	Normal

**Idiopathic**	Normal	Normal	Normal	Normal	Normal

To further complicate the diagnosis, HAE presentation may appear to be indistinguishable from idiopathic, acquired, allergic, or medication-induced AE if the family history of recurrent swelling is either unknown or absent – as in the case of spontaneous mutations. In addition, laboratory studies may prove inconclusive[20]. For example, a low/normal C1q level would not help the clinician distinguish between HAE and acquired angioedema. Therefore, clinicians may need to include genetic testing to make a definitive diagnosis. These tests are typically conducted in research laboratories since commercial laboratories do not yet offer the tests. It is important to note that not all mutations confer C1 INH deficiency. Clinicians can refer to the online database hae.biomembrane.hu as the most up-to-date source of documented mutations for comparison with results for their patients[21].

Idiopathic AE is the most common type of AE, affecting one in five people[22]. Idiopathic AE is the presence of ≥ 3 swelling episodes in six months to one year despite treatment trials[22]. Urticaria and pruritis will be present in half of cases[22]. Acquired AE is usually caused by a lymphoproliferative disorder or other autoimmune, infectious, or neoplastic disease[6]. Acquired AE has symptoms that closely resemble HAE. However, these symptoms do not emerge until middle life, helping distinguish acquired AE from HAE[23]. Caution should be exercised when interpreting laboratory studies for C1q since these levels may be normal in 25% of patients with acquired angioedema. A normal C1q result does not preclude a diagnosis of acquired angioedema[24,25].

ACE inhibitor use is the second most common cause of AE and may affect up to 6% of people taking these medications[26]. People of African descent are approximately 5 times more likely to experience this adverse event than people of European descent[26]. It typically presents in the first month of treatment, but may lie dormant for months or years[26]. The lips, tongue, and face are commonly affected sites, but the gastrointestinal tract may be involved[26]. ACE inhibitor-induced AE has a symptom pattern that is similar to HAE. This adverse event emerges when bradykinin degradation is slowed due to ACE inhibition[26]. The potential for increased availability of bradykinin makes this medication inappropriate for patients with HAE. Discontinuation remains the only treatment for ACE inhibitor-induced swelling.

In allergic AE, histamine is released via IgE-mediated mast cell degranulation in response to environmental allergens. In some patients, degranulation results from medication or contrast medium exposure. Autoantibody action involving IgE receptors on mast cells or basophils is also implicated in allergic AE.

## Available therapies

Available therapies are summarized in Table 2.

**Table 2:** Therapies for various angioedemas [6,22,26,27,29,33,34].

Angioedema type	Therapies*	
**HAE type 1** **HAE type 2** **Inherited angioedema with normal C1 inhibitor**	**Acute treatment** C1 inhibitor Kallikrein inhibitor Bradykinin β_2_-receptor inhibitor	**Prophylaxis** C1 inhibitor Danazol Antifibrinolytics are used in clinical practice. There are no approved therapies for medical/dental procedure prophylaxis.

**Acquired**	**Acute treatment** C1 inhibitor is the treatment of choice for laryngeal attacks. Doses greater than 1000 units may be required.	**Prophylaxis** Treatment of underlying disease. Antifibrinolytics are the first choice for long-term therapy, followed by attenuated androdgens.

**Angiotensin converting enzyme inhibitor induced**	ACE inhibitor discontinuation.	

**Idiopathic**	Antihistamines followed by glucocorticoids.	

*See text for United States vs. European approvals.

### Acute Treatment

Two acute treatments have recently become available in the United States. These medications provide an advance in available acute treatments, particularly because they are quick acting and directed at targets implicated in the pathophysiology of HAE. Although C1 INH has been used for 30 years in Europe and is currently marketed under the name Berinert P^®^, this plasma-derived C1 esterase inhibitor (human) was recently approved in the United States for the treatment of acute abdominal or facial HAE attacks in adults and adolescents. The medication is marketed under the name Berinert^® ^in the United States[27]. In a randomized, double-blind, placebo-controlled study that enrolled 125 adults and adolescents with HAE, patients were randomized to C1 inhibitor 10 units/kg, C1 inhibitor 20 units/kg, or placebo intravenous infusion[28]. Patients who received the 20 units/kg dose during an acute abdominal or facial HAE attack reported significantly shorter time to onset of relief compared with those receiving 10 units/kg or those receiving placebo. Berinert^® ^is supplied in 500 unit vials and administered intravenously at a dosage of 20 units per kg of body weight. The most common adverse reactions included subsequent HAE attack, abdominal pain, diarrhea, headache, muscle spasms, nausea, pain, and vomiting.

The kallikrein inhibitor, KALBITOR^®^, was also recently approved in the United States for the treatment of acute HAE attacks in patients 16 years and older[29]. Kallikrein inhibitor has not been approved for use by the European Medicines Agency. KALBITOR^® ^was studied in two double-blind, randomized, clinical trials that enrolled 143 individuals. In these trials, patients who were having an HAE attack at any anatomic location and who were experiencing at least one moderate to severe symptom were randomized to either 30 mg subcutaneous KALBITOR^® ^or placebo. Patients who received active medication reported statistically significant symptom improvement on a point-in-time symptom severity measure and a symptom response to treatment measure. More patients who received placebo required intervention for unresolved symptoms (50% vs. 33% of patients). KALBITOR^® ^is supplied in 10 mg vials. The approved 30 mg dosage is achieved via three subcutaneous injections, each containing 10 mg of the medication. Common adverse events (occurring in ≥ 3% of patients and higher than placebo) in these trials included headache, nausea, diarrhea, pyrexia, injection site reactions, and nasopharyngitis. Adverse event rates were higher when patients who received intravenous infusion of KALBITOR^® ^were included in the analysis. KALBITOR^® ^carries the risk of anaphylaxis. A black box warning is included in the prescribing information.

Firazyr^®^, icatibant (bradykinin β_2_-receptor inhibitor), is approved in Europe for acute treatment. Icatibant has been studied in two clinical trials[21,30]. In the randomized, double-blind, placebo-controlled FAST-1 trial, no treatment difference was observed between subcutaneous icatibant 30 mg and placebo in median time to symptom relief in adults with moderate to severe abdominal or cutaneous symptoms, although secondary endpoints showed benefit from icatibant administration. In the comparator-controlled, FAST-2 trial, subcutaneous icatibant 30 mg was shown to reduce time to onset of symptom relief compared with tranexamic acid in adults with abdominal or cutaneous symptoms. Subanalyses of these trials suggest that icatibant may be as effective for laryngeal swelling as for abdominal and cutaneous symptoms, with a time to first symptom improvement of ≤ 1 hour. Icatibant is supplied in a 30 mg dose and is administered subcutaneously. Common adverse events include mild injection-site reactions, abdominal pain, abnormal liver function tests, asthenia, dizziness, headache, nasal congestion, nausea, rash, and increased blood creatinine phosphokinase.

Rhucin^®^, recombinant C1 inhibitor, is currently under review by the European Medicines Agency for the acute treatment of HAE attacks.

### Prophylaxis

Routine prophylaxis has been shown to reduce the frequency and severity of HAE attacks. Danazol and C1 inhibitor are the only medications approved in the United States for prophylaxis. In Europe, attenuated androgens, antifibrinolytics, and C1 INH are used for prophylaxis. Although guidelines suggest that antifibrinolytics may be the least effective therapy[5]. The attenuated androgens, such as danazol, increase C1 inhibitor via the liver and carry the risk of long-term side effects[31]. An 84% reduction in the mean number of HAE attacks was associated with danazol prophylaxis in a retrospective study of 118 patients[32]. Nearly one-quarter of patients reported no HAE symptoms while on danazol therapy. Other patients continued to have attacks at a reduced frequency. Danazol is supplied in 200 mg, 100 mg, and 50 mg capsules and indicated for use in adults[33]. The 200 mg starting dose may be reduced to 100 mg in patients who do not experience significant breakthrough symptoms[33]. In clinical practice, danazol may be initially dosed as high as 600 mg/day and then titrated down to the lowest effective dose[9]. Adverse events were reported by nearly 80% of patients on danazol therapy and included weight gain, menstrual irregularities, female virilization, acne, headache, depression, and myalgia. Infrequent but serious cardiovascular and other adverse events were also reported.

The C1 inhibitor CINRYZE™ (C1 esterase inhibitor [human]) was recently approved in the United States for routine prophylaxis in adults and adolescents. In a randomized, double-blind, placebo-controlled, cross-over study, patients on active treatment experienced half as many HAE attacks as patients who received placebo[34]. Patients who experienced attacks while on C1 inhibitor therapy reported less severe and shorter attacks compared with those reported by patients on placebo. The study enrolled 24 patients with a history of ≥ 2 HAE attacks per month. Patients were randomized to either treatment arm: 1) 12 weeks of C1 inhibitor therapy, then 12 weeks of placebo or 2) 12 weeks of placebo, then 12 weeks of C1 inhibitor therapy. Twenty-two patients crossed over and received at least one dose of each study drug and completed the entire study. CINRYZE™ is administered intravenously every 3 to 4 days at a dose of 1000 units[34]. Upper respiratory infection, sinusitis, rash, and headache were the most commonly reported adverse reactions[34].

Although there are no approved treatments for medical or dental procedure prophylaxis, consensus guidelines recommend administration of C1 inhibitor one hour prior to procedures, with additional doses available at the time of the procedure, and as necessary thereafter[5]. The guidelines recommend high-dose danazol and fresh frozen plasma as second-line therapy[9].

## Summary

Advances in treatment for HAE have recently come to the market. These therapies have reduced potential for the long-term effects associated with attenuated androgen therapy and the adverse events associated with antifibrinolytics, both of which have been mainstays of treatment in the past. The newly available C1 inhibitor, kallikrein inhibitor, and bradykinin β_2_-receptor inhibitor work directly to reduce the massive bradykinin release in the contact plasma cascade that is thought to be the primary pathological mechanism in HAE. Appropriate prophylactic and acute use of these medications will help reduce patient risk for disability and death. Increased awareness of this disease and efficient steps in its differential diagnosis are essential to improving patient outcomes.

## Competing interests

Dr Eidelman has received clinical research funding from CSL Behring and Lev Pharmaceuticals and has received honoraria from Lev Pharmaceuticals, ViroPharma Incorporated, and CSL Behring.

## Authors' contributions

FJE contributed to the initial concept and design of the manuscript, provided critical review of the medical concepts and data presented, and approved the final version.

## Pre-publication history

The pre-publication history for this paper can be accessed here:

http://www.biomedcentral.com/1471-2326/10/3/prepub
